# Higher Physical Activity Is Associated With Lower Aortic Stiffness but Not With Central Blood Pressure: The ADDITION-Pro Study

**DOI:** 10.1097/MD.0000000000000485

**Published:** 2015-02-06

**Authors:** Anne Sofie Dam Laursen, Anne-Louise Smidt Hansen, Niels Wiinberg, Søren Brage, Annelli Sandbæk, Torsten Lauritzen, Daniel R. Witte, Marit Eika Jørgensen, Nanna Borup Johansen

**Affiliations:** From the Steno Diabetes Center, Gentofte, Denmark (ASDL, MEJ, NBJ); Department of Public Health, Section of General Practice, Faculty of Health Sciences, Aarhus University, Aarhus, Denmark (A-LSH, AS, TL); Department of Clinical Physiology, Frederiksberg Hospital, Copenhagen, Denmark (NW); MRC Epidemiology Unit, Institute of Metabolic Science, Addenbrooke's Hospital, Cambridge, United Kingdom (SB); Centre de Recherche Public de la Santé, Strassen, Luxembourg (DRW); and Danish Diabetes Academy, Odense, Denmark (NBJ).

## Abstract

Physical activity is associated with reduced cardiovascular disease risk. However, improvements in conventional risk factors due to physical activity do not explain its full benefit. Therefore, we examined associations of objectively measured physical activity energy expenditure and intensity with central hemodynamics to provide new insight into the link between physical activity and cardiovascular disease.

We analyzed data from 1816 Danes (median age: 66 years) without cardiovascular disease. Physical activity was estimated using combined accelerometry and heart rate monitoring. Aortic stiffness was assessed by applanation tonometry, as aortic pulse wave velocity, and central blood pressure was estimated from radial waveforms. Associations between physical activity energy expenditure and central hemodynamics were examined by linear regression. Furthermore, the consequence of substituting 1 hour sedentary behavior with 1 hour light or moderate-to-vigorous physical activity on central hemodynamics was examined.

Median physical activity energy expenditure was 28.0 kJ/kg/d (IQR: 19.8; 38.7). A 10 kJ/kg/d higher energy expenditure was associated with 0.75% lower aortic pulse wave velocity (CI: −1.47; −0.03). Associations with central systolic blood pressure and central pulse pressure were not statistically significant. We observed no difference in central hemodynamics when substituting 1 hour sedentary behavior with 1 hour light or moderate-to-vigorous physical activity.

In this relatively inactive population, higher physical activity energy expenditure was associated with lower aortic stiffness, while there was no statistically significant association between substitution of activity intensity and central hemodynamics. This suggests that lower aortic stiffness is one of a number of health benefits attributed to higher habitual physical activity.

## INTRODUCTION

Physical activity, defined as any bodily movement produced by skeletal muscle that results in energy expenditure above resting energy expenditure^1^ is associated with reduced cardiovascular disease risk.^2–4^ This beneficial effect of physical activity can be seen on a number of independent risk factors for cardiovascular disease including daytime ambulatory blood pressure,^5^ office blood pressure,^6^ and high-density lipoprotein cholesterol.^7^ However, improvement of these risk factors is insufficient to explain the positive effect of physical activity seen on cardiovascular events.^8^ Therefore, an investigation of markers that reflect the structural and functional abnormalities in the wall of the central elastic arteries, such as aortic stiffness and central blood pressure, might provide additional explanatory information about the relationship between physical activity and cardiovascular disease.

Studies investigating the relationship between physical activity and central hemodynamics are scarce, small and methodologically heterogeneous, and thus present inconsistent results. In observational studies, physical activity has been beneficially associated with various indices of arterial stiffness^9–12^ when comparing sedentary individuals with athletes and when comparing less active with more active individuals.^13–15^

The above studies estimated physical activity using different self-report and objective methods making them less comparable. Especially the use of self-reported physical activity instead of objective measures can lead to inconsistent results, as self-reported physical activity is only moderately correlated with objectively measured physical activity and has been found to both under- and overestimate physical activity, and measure low-intensity physical activity poorly.^16^

As of yet no other study has investigated the relationship of physical activity assessed by combined accelerometry and heart rate monitoring with central hemodynamics such as arterial stiffness and central blood pressure in a large sample of free-living adults. In order to better understand the physiological effects of physical activity on cardiovascular disease risk, we therefore aimed to investigate whether higher physical activity, expressed as physical activity energy expenditure (PAEE) and physical activity intensity are associated with lower arterial stiffness, central systolic blood pressure, and central pulse pressure in an elderly population.

## METHODS

### Design and Study Population

The study was a cross-sectional analysis based on data from a follow-up health examination (the ADDITION-PRO study^17^) of Danish adults, identified through a stepwise screening program for diabetes carried out in general practice in 2001 through 2006.^17^ The participants were 40 to 69 years of age and without known diabetes when entering the screening program. In 2009 to 2011, a follow-up health examination of a subset of those, who did not have diabetes at screening, was carried out. The selection and invitation procedure have previously been described.^17^ We invited 4188 persons of whom 2082 accepted and gave written informed consent. Because previous cardiovascular disease can impair the physical capability of the individual, participants with a self-reported history of cardiovascular disease (stroke, myocardial infarction, coronary artery bypass graft surgery, or percutaneous coronary intervention), and those where the absence could not be determined, were excluded from the analyses (n = 238) resulting in a study sample of 1844 individuals. Only participants with successful measurements of one or more of the outcome variables comprised the study sample (n = 1,816).

The study complied with the current Helsinki Declaration and was approved by the local ethics committee in the Central Denmark Region (no. 20000183).

### Health Assessments

The health assessments were carried out at 4 study centers in Denmark and performed by trained personnel following standard procedures. All participants fasted overnight before the study visit (≥8 h) except those who had developed diabetes since the screening program (n = 279), who were not asked to fast due to ethical reasons.

#### Central Hemodynamics

Arterial stiffness was assessed as aortic pulse wave velocity (aPWV), which is a measure of the average pulse wave velocity in the aorta and considered the best non-invasive method for assessing central arterial stiffness.^18^ The measurement of aPWV and central blood pressure was conducted by applanation tonometry using a SphygmoCor^®^ device (version 8, Actor Medical, West Ryde, NSW, Australia) and a high fidelity tonometer with the participant in the supine position. All measurements were undertaken at the right-hand side of the body. After 10 minutes rest, aPWV was assessed between the carotid and femoral arteries. The tonometer captured waveforms first at the carotid artery, then at femoral artery simultaneously with an electrocardiogram (ECG) recording using the intersecting tangent. The transit time was expressed as the mean of 10 pulse waves. A tape measure was used to measure the distance from the suprasternal notch to the carotid artery, whereas a caliper (Seca, Medical Scales and Measuring Systems, Hamburg, Germany) was used to measure the distance from the suprasternal notch to the femoral artery to avoid overestimation of the distance in individuals with accumulated abdominal fat. The net distance was expressed as the femoral-sternal notch distance subtracted the carotid-sternal notch distance and aPWV was expressed as travelled distance/transit time (m/s). Each participant had aPWV measured twice, and a third time if the difference between the 2 first measurements exceeded 0.5 m/s. The average of the 2 closest measurements was reported as aPWV.

Central systolic and diastolic blood pressure was estimated based on central waveforms calculated from peripheral waveforms recorded at the radial artery and calibrated by supine brachial systolic and diastolic blood pressure by a built-in generalized transfer function. Central pulse pressure was expressed as central systolic blood pressure subtracted central diastolic blood pressure. Supine brachial blood pressure was measured with an automated oscillometric blood pressure recorder (Omron M6 comfort, OMRON Healthcare Europe B.V., Hoofddorp, The Netherlands) after 10 minutes rest.

#### Physical Activity

The objectively measured physical activity data were collected using a combined accelerometer and heart rate monitor (Actiheart^®^, CamNTech Ldt., Cambridge, UK) as previously described.^19^ Briefly, during the health examination the device was placed on the participant's chest on 2 standard ECG electrodes. Subsequently, a submaximal step test was performed to account for differences in heart rate from person to person at given physical activity intensities.^20^ Disabled participants and participants with angina pectoris were excluded from performing the test. Participants were asked to wear the device for 1 week, not change their physical activity pattern during the period and to register non-wear time in a log. Electrodes were changed every second day to ensure a proper ECG signal during the whole week. The collected data were analyzed if there were ≥24 hours of complete data.

First, the collected minute-by-minute accelerometer and heart rate data were checked for noisy heart rate and non-wear time^21^ after which, physical activity measures were derived using a branched equation model.^22^ The heart rate to PAEE relationship was calibrated with data obtained from each participant's step test. For participants who did not perform the step test, the heart rate to PAEE relationship was estimated by a group calibration, derived based on calibration coefficients from all complete individual calibrations.^20^ The group calibration was applied for 463 participants whereas 941 participants had individually calibrated estimates (total n = 1404).

Physical activity intensity was expressed as multiples of resting metabolic rate (METs) and calculated taking into account the basal metabolic rate of the individual which was calculated using the Oxford prediction equations.^23^ The physical activity intensity groups were defined as; sedentary behavior (<1.5 METs), light physical activity (≥1.5–3.0 METs), moderate-to-vigorous physical activity (MVPA) (>3.0 METs). The time spent in physical activity intensity groups per hour was calculated based on the minute-by-minute PAEE measurements. Finally, the physical activity measures were summarized to daily measures (kJ/kg/day and hours/day), whilst minimizing diurnal information bias.

#### Other Health Assessments

Waist circumference was measured with a D-Loop tape while the participant was in standing position. It was measured to the nearest millimeter at the mid-point between the lower coastal margin and anterior superior iliac crest. Measurements were completed twice and a third time if the difference between the 2 first measurements exceeded 3 cm. The mean value of the 2 closest measurements constituted the value for waist circumference. Plasma triglycerides were collected in lithium-heparinized tubes and the content was quantified by reflectance spectrophotometry.^17^ Lifestyle and general health characteristics including smoking, drinking habits, medication use and previous cardiovascular disease were collected via a general health questionnaire filled out prior to the health examination and checked for completeness at the visit.

### Statistics

First, we modelled aPWV, central systolic blood pressure and central pulse pressure as linear regression functions of total PAEE. Second, the same outcomes were modelled with time spent in light and MVPA as primary explanatory variables with sedentary behavior being the reference. We thus investigated the difference in the outcome variables associated with spending less time in sedentary behavior and more time in light and MVPA.

Multiple imputation of missing values (Table 1) was done in order to accommodate the potential biases introduced by the missingness as well as the decreased power. All regression analyses were performed on imputed data. The imputations were generated using fully conditional specification^24^ and 50 imputed data sets were generated. The analyses were then performed on each independent data set and ultimately combined in a mean parameter estimate for each individual analysis using Rubin's rules.^25^ Imputed variables included all variables used in the linear regression models as well as variables potentially related to these.

**TABLE 1:**
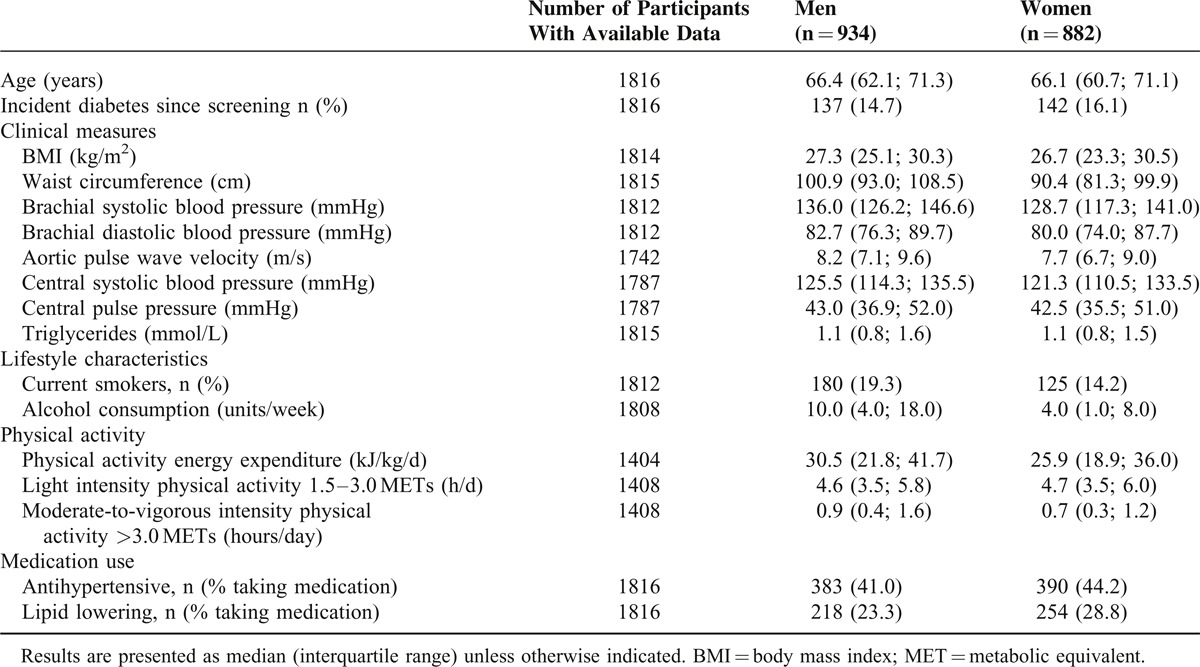
Participant Characteristics by Sex

The associations were initially explored with inclusion of interaction terms for sex and PAEE, and diabetes and PAEE. To better approximate normally distributed residuals, aortic PWV was logarithmically transformed. The regression models were successively adjusted for age, sex, resting heart rate at the time of aPWV and central blood pressure measurement, (as well as mean blood pressure in analyses with aPWV) (model 1), waist circumference (model 2), smoking, plasma triglycerides, incident diabetes, lipid lowering and antihypertensive medication (model3). In order to assess the robustness of the results, a sensitivity analysis was conducted by repeating the linear regression analyses only on participants without diabetes, not taking any antihypertensive or lipid lowering medication. For all statistical analyses SAS 9.3 (SAS Institute, Cary, NC) was used.

## RESULTS

Median PAEE was 30.6 and 25.9 kJ/kg/d for men and women, respectively. In general, men had a worse cardiovascular risk profile compared with women; they had higher BMI, brachial and central blood pressure and aPWV. Moreover, they had a higher alcohol intake and were more likely to be smokers (Table 1).

### Associations Between Physical Activity and Central Hemodynamics

#### PAEE

**FIGURE 1:**
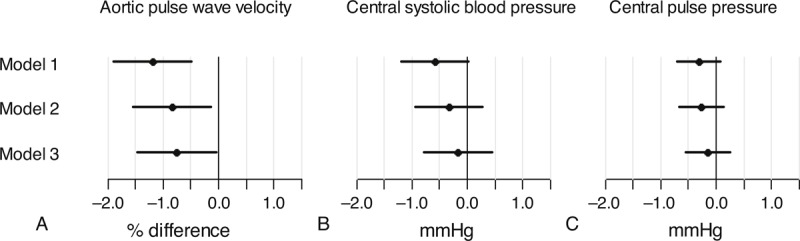
Difference in central hemodynamics by a difference of 10 kJ/kg/d in PAEE. Model 1 adjusted for sex, age, heart rate at the time of measurement and mean blood pressure (only analyses with aPWV). Model 2 = model 1 adjusted for waist circumference. Model 3 = model 2 adjusted for smoking, triglycerides, antihypertensive and lipid lowering treatment and incident diabetes.

**FIGURE 2:**
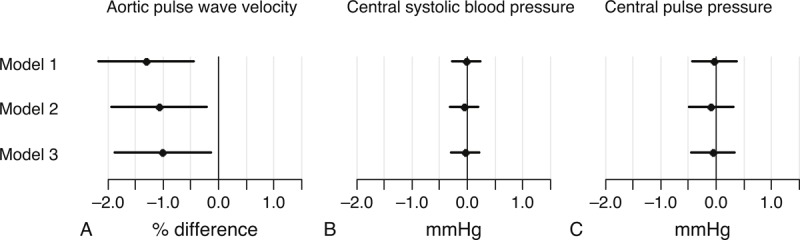
Difference in central hemodynamics by a difference of 10 kJ/kg/d in PAEE in participants without type 2 diabetes and not taking antihypertensive or lipid lowering medication. Model 1 adjusted for sex, age, heart rate at the time of measurement and mean blood pressure (only analyses with aPWV). Model 2 = model 1 adjusted for waist circumference. Model 3 = model 2 adjusted for smoking and triglycerides.

Initial regression analyses exploring an interaction between sex and PAEE and an interaction between diabetes and PAEE in relation to central hemodynamics revealed no interactions, thus the results are presented for the whole sample. A higher PAEE of 10 kJ/kg/d was associated with a 1.20% (95% CI: −1.90; −0.50) lower aPWV when adjusting for sex, age, heart rate and mean blood pressure. The estimated difference attenuated with additional adjustment, yielding a 0.75% (95% CI: −1.47; −0.03) lower aPWV (Figure 1a). Central systolic blood pressure and central pulse pressure were not associated with a difference in PAEE (Figure 1b and 1c). The sensitivity analyses, where participants with known diabetes and those taking any antihypertensive or lipid lowering medication were excluded, yielded slightly higher estimates for aPWV (Figure 2a). For central systolic blood pressure and central pulse pressure, the estimates were attenuated toward zero compared to the results from the full sample (Figure 2b and 2c).

#### Physical Activity Intensities

**FIGURE 3:**
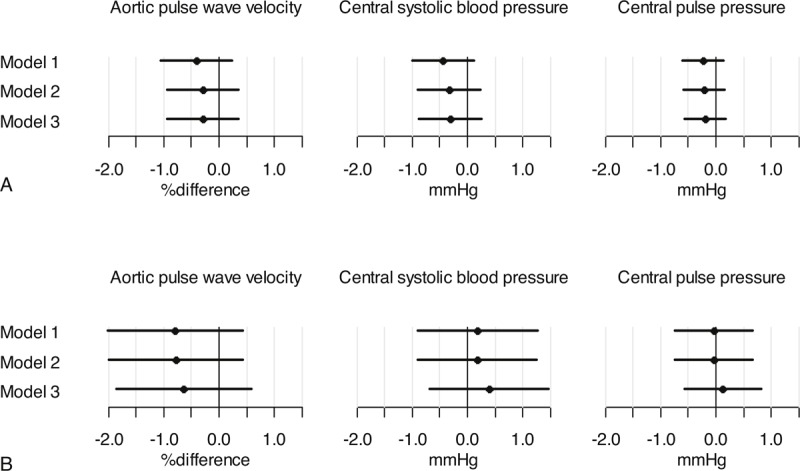
Substitution of sedentary behavior with light physical activity or MVPA. (a) One hour of sedentary behavior substituted with 1 hour light physical activity. (b) One hour of sedentary behavior substituted with 1 hour MVPA. Model 1 adjusted for sex, age, heart rate at the time of measurement and mean blood pressure (only analyses with aPWV). Model 2 = model 1 adjusted for waist circumference. Model 3 = model 2 adjusted for smoking, triglycerides, antihypertensive and lipid lowering treatment and incident diabetes.

**FIGURE 4:**
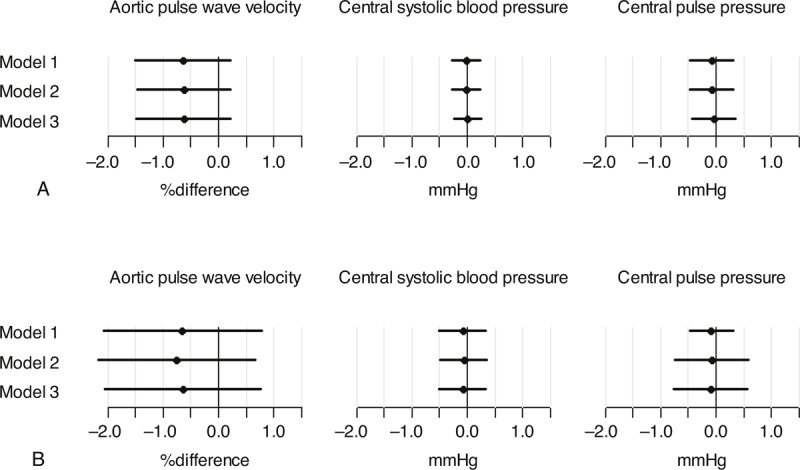
Substitution of sedentary behavior with light physical activity or MVPA in participants without type 2 diabetes and not taking antihypertensive or lipid lowering medication. (a) One hour of sedentary behavior substituted with 1 hour light physical activity. (b) One hour of sedentary behavior substituted with 1 hour MVPA. Model 1 adjusted for sex, age, heart rate at the time of measurement and mean blood pressure (only analyses with aPWV). Model 2 = model 1 adjusted for waist circumference. Model 3 = model 2 adjusted for smoking and triglycerides.

We investigated the difference in aPWV, central systolic blood pressure and central pulse pressure associated with spending 1 hour less in sedentary behavior and instead 1 more in either light or MVPA. We found no associations for either outcome with substitution of sedentary time with physical activity (Figures 3 and 4).

## DISCUSSION

### Discussion of the Main Results

In this large-scale observational study with detailed measurements of physical activity, aortic stiffness and central blood pressure we found that a 10 kJ/kg higher daily PAEE was associated with a 0.75% lower aPWV in middle-aged and elderly adults without cardiovascular disease. As an example, we can compare 2 participants who are similar in all aspects but PAEE and aPWV; if one has a PAEE of 28 kJ/kg/d and an aPWV of 8.0 m/s, the other would have an aPWV of 7.94 m/s if having a PAEE of 38 kJ/kg/d.

A difference in PAEE of 10 kJ/kg equals 800 kJ per day for a person weighing 80 kg (mean weight in our study population) and corresponds to approximately 1 hour of light walking or half an hour of light bicycle riding.^26,27^ Although previous studies have examined the relationship between physical activity and central hemodynamics, this study is the first to investigate associations of physical activity measured by combined heart rate and activity monitoring with aPWV and central blood pressure in middle-aged and elderly adults. The large variability in assessment methods makes it difficult to directly compare inferences drawn from previous studies. Early research investigating physical activity and arterial stiffness, identified an association between higher habitual physical activity level and lower aPWV.^11,12^ Results from an observational study comparing sedentary individuals with aerobically trained individuals support this notion.^10^ Similarly, higher pedometer-assessed physical activity (steps/day) has been associated with lower aPWV in Swedish adults with type 2 diabetes^13^ and elderly Japanese adults.^14^ In contrast, accelerometer-assessed physical activity was not associated with aPWV in a small study conducted in young to middle-aged adults at low cardiovascular risk.^28^ The lack of an association could be due to the population being younger than ours and at low-risk, consequently having less variation in aPWV. It could also be because accelerometry estimates physical activity less accurately compared to combined accelerometer and heart rate monitoring, and thus potentially yields smaller variation in estimated physical activity.

A meta-analysis, although including various methods of aPWV, found a relative risk of 1.14 (95% CI: 1.09; 1.20) and 1.15 (95% CI: 1.09; 1.21) for cardiovascular events and cardiovascular death respectively, with an aPWV increase of 1 m/s. For a participant in our study with aPWV of 8.0 m/s, a 0.75% lower aPWV (0.06 m/s) would then correspond to a risk reduction of 0.84%, a risk reduction, which might not be clinically relevant for the individual but could influence population risk.

When conducting sensitivity analyses by excluding participants with diabetes or taking antihypertensive or lipid lowering medication, the parameter estimates increased for aPWV. This indicates that higher total daily physical activity is associated with less aortic stiffness in healthy older adults but to a lesser extent those who have impaired cardio-metabolic function. From these results it can be proposed that daily physical activity might be an integrated part of a lifestyle that promotes healthy aging, although reversed causality cannot be ruled out. For central blood pressure, the estimates attenuated toward zero with narrow confidence intervals emphasizing that in the healthiest participants, central blood pressure and physical activity are not associated.

In our study population, we observed no association between substitution of time spent in sedentary behavior with light physical activity or MVPA and the hemodynamic outcomes. In contrast, Gando et al^29^ demonstrated that in elderly adults, aPWV is independently predicted by time spent in light and moderate physical activity. This conflict could potentially be explained by methodological differences; Gando et al assessed physical activity by accelerometry alone and did not adjust for the same confounding factors we did. However, in our study population physical activity was primarily of light intensity and only little time was spent in moderate or vigorous physical activity. Thus, too little variation and range in physical activity intensity could also explain why total PAEE was associated with aPWV but not when differentiating between activity intensity, despite not being able to account for the increase in PAEE when substituting sedentary behavior with light physical activity or MVPA.

The sensitivity analyses for substituting activity time did not change the results for aPWV markedly but the estimates for central blood pressure attenuated towards zero with narrow confidence intervals again indicating that central blood pressure is not associated with physical activity in the healthiest participants.

We found median PAEE of 30.6 and 25.9 kJ/kg/d for men and women, respectively, which is comparatively lower than that of previous reports also measuring PAEE by combined accelerometry and heart rate monitoring.^30^ In another Danish population, median PAEE was 42.1 and 38.0 kJ/kg/d for men and women, respectively.^30^ In the UK it was 36.4 and 34.1 kJ/kg/d in men and women respectively.^30^ These differences could be due to a slightly higher age in our sample and/or the fact that our sample primarily consists of high-risk individuals.

None of our analyses investigating central blood pressure revealed an association with physical activity. This is in contrast to Hefferman and colleagues^31^ who found associations of both self-reported physical activity and sitting time with components of central blood pressure in young adults. Conversely, self-reported television time (as a proxy for sitting time) was not associated with central systolic blood pressure in a population more similar in age to our participants when adjusted for relevant confounders.^32^

Finally, despite heterogeneity in methodology, current evidence suggests that physical activity benefits aPWV. Our study has contributed to strengthen this evidence but a causal dose-response relationship has yet to be established; this should ideally be examined in a randomized controlled trial of cardiovascular disease risk prevention through physical activity at different levels.

### Strengths and Limitations

A clear strength of this study is the large sample size as most studies with similar aims have based their results on much smaller samples. The use of an objective measure for the assessment of physical activity further strengthens the accuracy and reliability of our data. For logistical and financial reasons, large scale observational studies have traditionally estimated physical activity by self-report but this might lead to spurious results as self-report measures have shown to yield both lower and higher estimates when compared to objective and direct measures of physical activity.^16,33^ The use of accelerometry in combination with heart rate measurements for the estimation of PAEE has been shown to provide more accurate estimates than either measure alone in both experimental and free-living settings,^34–36^ and individual calibration further improves the estimation.^34,35^ Although objective, the derived estimate of PAEE used in the current study is not a direct measure of energy expenditure, but unlike directly measured energy expenditure this is a feasible method to assess daily living physical activity at a population level.

Parameters of central hemodynamics were measured according to the 2006 guidelines,^18^ yielding low inter and intra-observer variability as previously demonstrated in one of our study centers.^37^ By the use of a caliper instead of a tape measure, we avoided overestimation of the travelled distance and thereby aPWV in overweight and obese individuals. The calibration of the radial pressure waveform with brachial blood pressure and the use of a generalized transfer function to estimate central blood pressure are sources of bias and could theoretically have affected our findings for central blood pressure.

Although we adjusted for a number of potential confounders, residual confounding could still be an issue. However, once adjusted for waist circumference, the results for aPWV did not change markedly with any further adjustment, indicating that waist circumference might capture a number of factors related to the metabolic risk profile, which are related to both arterial stiffness and physical activity.

Missing data in observational studies is a frequently encountered problem that can never fully be avoided. Omitting observations with missing values as is the case with complete-case analyses induces risk of biased results. Using multiple imputation by fully conditional specification to accommodate missing values has been demonstrated to produce less bias than other incomplete-data methods^38,39^ and is an appropriate imputation method when the missing data are both categorical and continuous.^24^

Due to the cross-sectional study design, we are unable to draw causal inferences. However, our results demonstrated that even a modestly higher overall PAEE was associated with less arterial stiffness.

## CONCLUSION

To conclude, in an elderly population free of cardiovascular disease and with relatively low physical activity, we found that a higher PAEE was associated with a lower level of aortic stiffness, whereas the intensity of physical activity was not statistically significantly related to aortic stiffness. In contrast, we observed no associations between PAEE or physical activity intensity and central blood pressure. These results suggest that lower aortic stiffness is one of a number of health benefits attributed to higher habitual physical activity and might thus contribute to the accumulated effect of physical activity on lowering cardiovascular disease risk. Nonetheless, it remains to be determined if an increase in habitual physical activity will reduce arterial stiffness and by how much, and whether this in turn will cause a reduction in cardiovascular disease morbidity and mortality.

## SOURCES OF FUNDING

ADDITION-Denmark was supported by the National Health Services in the counties of Copenhagen, Aarhus, Ringkøbing, Ribe, and South Jutland in Denmark; the Danish Council for Strategic Research; the Danish Research Foundation for General Practice; Novo Nordisk Foundation; the Danish Center for Evaluation and Health Technology Assessment; the Diabetes Fund of the National Board of Health; the Danish Medical Research Council; and the Aarhus University Research Foundation. The trial has been given unrestricted grants from Novo Nordisk AS, Novo Nordisk Scandinavia AB, Novo Nordisk UK, ASTRA Denmark, Pfizer Denmark, GlaxoSmithKline Pharma Denmark, Servier Denmark A/S, and HemoCue Denmark A/S. Parts of the grants from Novo Nordisk Foundation, Danish Council for Strategic Research, and Novo Nordisk were transferred to the other European centers.

The ADDITION-PRO study is funded by an unrestricted grant from the European Foundation for the Study of Diabetes/Pfizer for Research into Cardiovascular Disease Risk Reduction in Patients with Diabetes (74550801) and internal research and equipment funds at Steno Diabetes Center. NBJ is funded by the Danish Diabetes Academy supported by the Novo Nordisk Foundation.
